# Circulating Tumour DNA After Neoadjuvant Therapy in Non-Metastatic Colon Cancer: A Systematic Review and Implications for Surgical Decision-Making

**DOI:** 10.3390/cancers18050815

**Published:** 2026-03-03

**Authors:** Mahmoud M. Salama, Charles Eddershaw, Hugo C. Temperley, Arvin Kumar Perthiani, John O. Larkin, Brian J. Mehigan, Dara O. Kavanagh, Paul H. McCormick, David Gallagher, Charles Gillham, Emily Harrold, Michael E. Kelly

**Affiliations:** 1 School of Medicine, Trinity College Dublin, D02 PN40 Dublin, Ireland; salamam@tcd.ie; 2 Trinity St James Cancer Institute, St James Hospital, D08 NHY1 Dublin, Ireland; 3 Department of Surgery, Royal College of Surgeons in Ireland, 123 St Stephen’s Green, D02 YN77 Dublin, Ireland; 4 Department of Radiology, St James Hospital, D08 NHY1 Dublin, Ireland; 5 Department of Colorectal Surgery, St James Hospital, D08 NHY1 Dublin, Ireland; 6 Department of Colorectal Surgery, Tallaght University Hospital, D24 NR0A Dublin, Ireland; 7 Department of Oncology, St James Hospital, D08 NHY1 Dublin, Ireland; 8 Department of Radiation Oncology, St James Hospital, D08 NHY1 Dublin, Ireland

**Keywords:** colon cancer, immunotherapy, dMMR, circulating tumour DNA, ctDNA, watch and wait, non-operative management

## Abstract

Introduction: Neoadjuvant systemic and immunotherapy strategies in non-metastatic colon cancer have demonstrated high pathological response rates, raising interest in surgery-sparing approaches. Circulating tumour DNA (ctDNA) is an emerging biomarker for treatment response and minimal residual disease, but its role in guiding surgical omission in colon cancer remains unclear. This systematic review evaluates the diagnostic and prognostic accuracy of ctDNA in predicting pathological response following neoadjuvant therapy in non-metastatic colon cancer. Methods: A systematic review was conducted in accordance with PRISMA guidelines. PubMed, Embase/MEDLINE, Scopus, and the Cochrane Register were searched from inception to 21 October 2025. Eligible studies included adults with non-metastatic colon cancer treated with neoadjuvant therapy who had serial ctDNA assessment prior to surgery. Results: Three cohort studies comprising 100 patients met inclusion criteria. Baseline ctDNA detection ranged from 42% to 84%. Across studies, ctDNA clearance following neoadjuvant therapy was consistently associated with major pathological response or pathological complete response, whereas persistent ctDNA strongly predicted residual viable tumour at resection. In the largest prospective cohort, 5 of 26 patients (19%) achieved ctDNA clearance prior to surgery; all were pathological responders, while 19 of 26 patients (73%) with persistent ctDNA demonstrated no pathological response. No study reported pathological complete response in the presence of persistently positive ctDNA. No prospective trial formally evaluated ctDNA-guided surgical omission. Conclusions: Current evidence does not support the use of ctDNA alone to guide omission of surgery after neoadjuvant therapy in non-metastatic colon cancer—even in patients who show complete pathological response. While persistent ctDNA reliably identifies patients with residual disease, ctDNA clearance lacks sufficient positive predictive value to safely forego surgery. Prospective trials with standardised ctDNA platforms and predefined non-operative management protocols are required before ctDNA-guided organ preservation can be recommended.

## Simple Summary

Surgical resection remains the standard of care for patients with non-metastatic colon cancer. Recent advances in molecular testing of colon cancer have raised the question of whether neoadjuvant therapy may be an option for the management of these malignancies. Blood-based assays that detect tumour-derived genetic material are increasingly being investigated as minimally invasive tools to assess treatment response and residual disease. There is growing interest in whether clearance of tumour-derived material from the blood after neoadjuvant therapy could support consideration of non-operative management in select patients. This study systematically reviews the available evidence examining circulating tumour DNA dynamics in this setting. The findings indicate that, while changes in blood-based tumour markers may reflect pathological response, the current evidence base is limited in size and heterogeneous in methodology. Consequently, there is insufficient evidence to support the routine use of these assays to guide omission of surgery in clinical practice. These results define important evidence gaps and highlight priorities for future prospective trials.

## 1. Introduction

Colorectal cancer (CRC) is the fourth most common cancer and second leading cause of cancer death worldwide with an estimated two million new cases diagnosed each year [1]. Recent trends show an increase in incidence, especially among younger adults, and CRC is now the leading cause of death in men under age 50 [2]. The global burden of colorectal cancer is expected to increase by 60% by the year 2030 [2].

The current standard of care for non-metastatic colon cancer is oncological resection followed by adjuvant chemotherapy in select cases [3]. In patients undergoing surgery, bowel resection remains a complex procedure with significant morbidity. Furthermore, adjuvant chemotherapy is associated with a significant adverse side effect profile. Recent advances in molecular understanding of CRC have led to the concept of neoadjuvant immunotherapy for a select cohort of CRC patients with microsatellite instability-high (MSI-H) or mismatch repair-deficient tumours (dMMR) [4,5]. Trials such as the NICHE-2 study reported high rates of complete pathological response in patients with MSI-H/MMR colon cancer after neoadjuvant immune checkpoint inhibition [6]. The role of surgery-sparing treatment has been extensively studied in rectal tumours, and the so-called “watch-and-wait” approach is now considered an acceptable standard of care in select patients with rectal cancer [7]. However, given recent trial results showing a high proportion of complete clinical response after neoadjuvant immunotherapy, the question now is whether surgery is necessary at all in patients with localised colonic tumours who achieve a complete response to neoadjuvant therapy.

A reliable biomarker that can predict which patients will remain in remission and which still harbour disease that has not been fully managed is essential. Circulating tumour DNA (ctDNA) is a tumour-derived, single- or double-stranded DNA fragment secreted by tumour cells into the bloodstream. It has recently emerged as a promising biomarker across various cancer types, most notably in colorectal cancer [8]. In patients with colorectal cancer, ctDNA has a well-validated ability to detect minimal residual disease (MRD), monitor treatment response, and stratify patients into risk groups for recurrence [9]. Furthermore, the DYNAMIC study suggested that colon cancer patients with negative ctDNA after surgery may safely forego adjuvant treatment without compromising recurrence-free survival [10]. Emerging evidence in rectal cancer indicates that ctDNA negativity after total neoadjuvant therapy (TNT) predicts both clinical and pathological complete response and may therefore be used as a diagnostic tool to identify patients who may benefit from non-operative management (NOM) [11,12]—reliant on complete disease clearance on endoscopy, radiological imaging (CT scans) and histology from biopsies of the site. The same utility of ctDNA in the neoadjuvant setting in colon cancer has yet to be explored.

However, while ctDNA is recognised as a prognostic marker, its role in guiding surgical decision-making remains unclear. Specifically, it is uncertain whether current evidence supports the use of ctDNA beyond describing treatment response and justifying NOM in patients who achieve apparent complete response.

Our study aims to evaluate the prognostic accuracy and clinical utility of ctDNA in predicting both clinical and pathologic complete response rates in adults with colon adenocarcinoma treated with neoadjuvant therapy. Our objective is to inform the use of ctDNA to identify patients who can safely undergo NOM with acceptable oncologic outcomes.

## 2. Methods

We designed this systematic review and reported the results in compliance with the Preferred Reporting Items for Systematic Reviews and Meta-Analyses (PRISMA) guidelines [13] (see Supplementary Materials). These review methods were established prior to the conduct of the review following agreement between all study authors. This review was prospectively registered with the international Prospective Register of Systematic Reviews (PROSPERO Registration ID: CRD420251242673).

### 2.1. Search Strategy

We performed an electronic search of PubMed, Embase/Medline, Scopus and Cochrane Register of Controlled Trials databases. Additionally, the obtained articles’ references were screened for any relevant publications that may have been missed. The final search was conducted on 21 October 2025. A comprehensive search was carried out by combining the following search terms with AND/OR Boolean operators: (circulating tumour DNA OR circulating tumor DNA OR cell free DNA OR ctDNA or cfDNA OR liquid biopsy or liquid biopsies OR tumour derived nucleic acid or cell free circulating DNA or cfcDNA or cftDNA) AND (colon cancer OR colonic cancer OR colon malignancy OR colonic malignancy OR colon carcinoma OR colon adenocarcinoma OR colon neoplasm OR colonic neoplasm OR colorectal cancer OR colorectal malignancy OR colonic tumour OR colonic tumor OR colonic neoplasm) AND (clinical response OR pathological response OR complete clinical response OR complete pathological response OR treatment response OR minimal residual disease OR MRD OR cCR OR cPR OR full response OR disease free). No limit was placed on date of publication. Articles were limited to those with available English translation.

### 2.2. Inclusion Criteria

Studies were included if they met the following criteria: patients were at least 18 years of age with non-metastatic colon cancer, reported on the use of neoadjuvant treatment strategies, and reported on ctDNA levels in these patients. The study designs included randomised controlled trials, cohort studies, and large case series (>5 patients) that met all the key parameters outlined above. Studies in which patients underwent surgery and that reported on ctDNA levels and preoperative histological/endoscopic pathological status were included.

### 2.3. Exclusion Criteria

Studies were excluded if they reported on patients with metastatic disease, cancers other than colonic and where ctDNA levels could not be matched to the various points in the treatment journey. Studies were excluded if they were small case series (<5 patients), case reports or conference abstracts. Studies focusing on utility of ctDNA in an adjuvant setting and where preoperative ctDNA levels and disease status were lacking were excluded.

### 2.4. Study Selection

All extracted studies were uploaded to COVIDENCE software [14] which automatically removed all duplicate studies. Two independent reviewers (MMS and CE) independently screened all relevant titles and abstracts. Any disagreements at the screening stage were settled by a third reviewer (AKP). Relevant studies were sought for full text review and again each reviewer analysed these to ensure they met the above criteria. All disagreements at this stage were settled by discussion between all three reviewers (MMS, CE and AKP) until a final verdict was reached.

### 2.5. Data Extraction

Relevant data, which were extracted, included study design and setting, number of included patients and tumour characteristics (including dMMR status), neoadjuvant treatment regimen, ctDNA assay type and timing, ctDNA response, and histological and/or surgical pathology outcomes—i.e., pathological complete vs. near complete vs. no response to neoadjuvant treatment regimen. Extracted results were collected on a formatted Excel sheet by two reviewers (MMS and CE) independently and checked by a third reviewer (HT) to ensure correct data entry.

The Newcastle–Ottawa scale was used to assess the risk of bias in the included studies [15].

### 2.6. Statistical Analysis

A formal quantitative meta-analysis was not undertaken, as substantial clinical and methodological heterogeneity across the included studies precluded meaningful pooling of effect estimates. Key sources of heterogeneity included differences in stage distribution and tumour biology (including mismatch repair status), neoadjuvant treatment regimens, ctDNA assay platforms and thresholds, pathological response definitions, and the timing of posttreatment blood sampling. In addition, incomplete reporting of subgroup-specific outcomes and small sample sizes further limited comparability and the reliability of any pooled estimates.

### 2.7. Definitions and Outcome Harmonisation

For the purposes of this systematic review, standardised operational definitions were prespecified to maximise cross-study comparability:

ctDNA status and dynamics: Baseline ctDNA positivity was defined as detectable ctDNA prior to initiation of neoadjuvant therapy according to each study’s assay-specific threshold for detection. ctDNA clearance (i.e., converters) was defined strictly as conversion from ctDNA-positive at baseline to ctDNA-undetectable in the preoperative (postneoadjuvant) sample. Patients who remained ctDNA-positive preoperatively were labelled as persistently positive (non-converters). Patients who were ctDNA-negative at baseline and remained negative preoperatively were categorised separately as persistently negative and were not included in analyses of ctDNA clearance, given the absence of demonstrable molecular response. Where studies did not report baseline ctDNA status or did not permit separation of these categories, we excluded them from our analysis.

Pathological response: Given heterogeneity in pathological response grading systems across studies, pathological response was harmonised into clinically meaningful strata where possible: pathological complete response (pCR: no residual viable tumour), major pathological response (MPR: <10% residual viable tumour or equivalent definitions where reported), and lesser degrees of regression or no response. Where original studies reported alternative grading systems (e.g., modified Ryan score), these were mapped to the above categories where feasible. Where mapping was not possible due to insufficient reporting granularity, results were summarised descriptively without cross-study quantitative comparison.

Timing of ctDNA sampling: Postneoadjuvant ctDNA assessment was defined as the last blood sample obtained following completion of neoadjuvant therapy and prior to surgical resection. Given variability in sampling windows across studies, the timing of ctDNA collection relative to treatment completion and surgery was extracted and reported study-by-study where available. Recognising the short half-life of ctDNA and the potential impact of sampling timing on false-negative rates, this heterogeneity was considered a key source of methodological variability and precluded quantitative pooling of results.

## 3. Results

### 3.1. Study Selection

**Figure 1:**
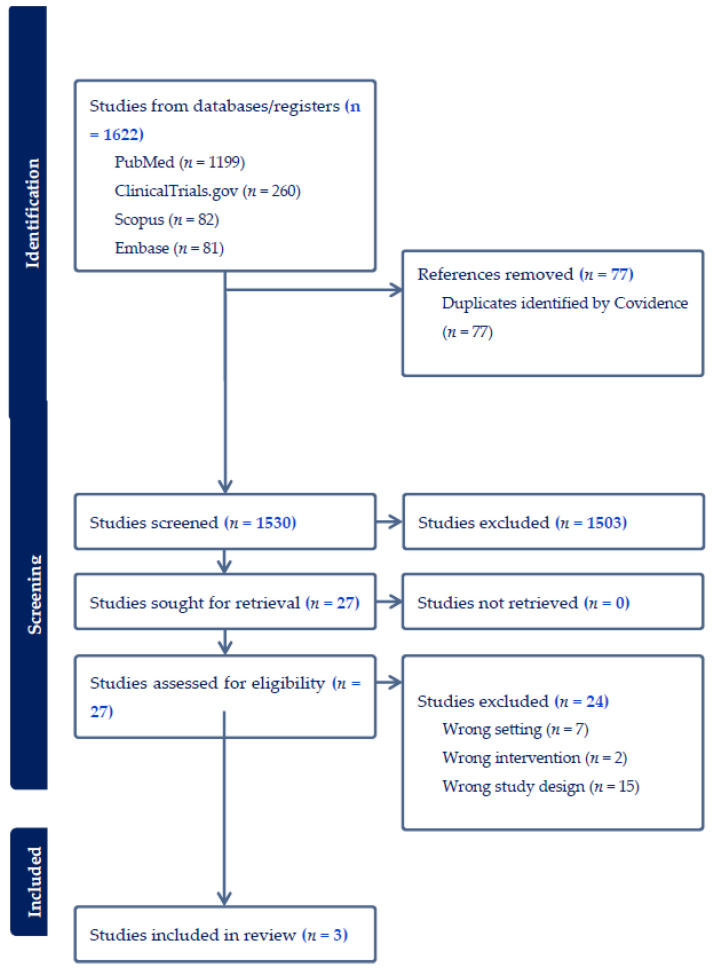
PRISMA Flowchart for Study Selection.

Our study selection process is presented in the PRISMA flowchart in Figure 1. Our search yielded 1622 records. After removing of 77 duplicates, 1530 records were manually screened by title and abstract. Twenty-seven articles passed initial screening and were sought for full text review. Of these, only three studies [16,17,18] met our predefined eligibility criteria and were included in the qualitative synthesis.

### 3.2. Bias Assessment

**Figure 2:**
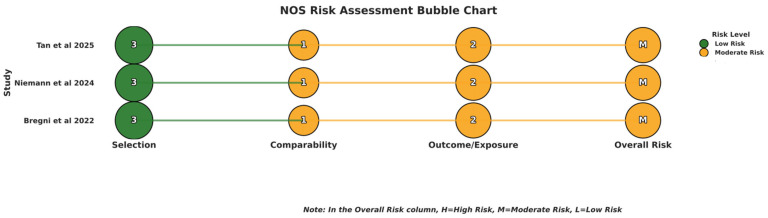
Risk of bias assessment using the Newcastle–Ottawa Scale [15,16,17,18].

Using the Newcastle–Ottawa tool for risk of bias assessment in cohort studies [15], all three studies had moderate risk of overall bias, each scoring a total of six stars (Figure 2).

### 3.3. Study Characteristics

Our three included studies were all translational cohort analyses [16,17,18] evaluating the role of ctDNA in colon cancer. All studies included serial ctDNA measurements obtained at baseline, after neoadjuvant treatment and prior to surgical resection (if patients proceeded to surgery).

There was a noted variation in the baseline type of colon cancer across all three studies. Two studies investigated the role of ctDNA in the neoadjuvant setting of colon cancer, with no mention of MMR status [16,17]. Neoadjuvant treatment in both studies involved systemic chemotherapy or immunotherapy-based regimens (although the breakdown of patients with pMMR or dMMR was not provided). Of note, one of these studies (Niemann et al., [17]) included a population of colon and rectal cancers, but only data pertaining to colon cancer were reported. The final included study assessed patients with pMMR colon tumours receiving immunotherapy [18].

All included studies had small sample sizes of patients eligible for investigation in our study, ranging from 4 eligible patients [17] to 60 patients [16], reflecting the early exploratory nature of these studies. Overall, 94 patients from all three studies were included in our analysis. All studies mandated surgical resection following neoadjuvant treatment. However, we analysed only patients with baseline and postneoadjuvant ctDNA levels available before surgical resection. Table 1 below outlines the various studies and their population characteristics.

Methodologies for ctDNA assessment also varied across the included studies. Methylation-based droplet digital PCR was used in one study [16], while tumour-informed next-generation sequencing platforms were used in the other two [17,18]. All studies obtained ctDNA samples at consistent and clinically relevant time points, including prior to initiation of neoadjuvant treatment regimens, after neoadjuvant treatment, and postsurgery.

### 3.4. ctDNA Detection and Dynamics During Neoadjuvant Therapy

Across all included studies, baseline ctDNA was detectable in the majority of patients prior to initiation of neoadjuvant therapy. Detection rates ranged from approximately 42% [16] to 83% [18] depending on tumour biology and ctDNA assay methodology. Table 2 outlines variation in ctDNA level and correlation with pathological status when available.

Bregni et al., 2022 [16] included 80 patients with stage II–III colon cancer who received one cycle of neoadjuvant FOLFOX chemotherapy. Of these, 60 had assessable ctDNA at baseline. Of these, 25 were ctDNA-positive (42%) prior to neoadjuvant therapy. Of the 80 patients, only 76 had histology samples available postoperatively. The authors reported that only seven of these patients had some form of tumour regression, with the rest having no or poor response to neoadjuvant chemotherapy. The study showed that positive ctDNA at baseline or at any single preoperative time point was not independently associated with disease-free survival. However, patients who demonstrated an increase in ctDNA levels between baseline and surgery showed a trend toward lower 5-year disease-free survival (HR 3.66).

Niemann et al., 2024 [17] was a retrospective cohort study assessing 24 patients with stage III and IV colorectal cancer. Of these, four patients had non-metastatic colon cancer (all stage III) with detectable ctDNA at baseline and before neoadjuvant treatment. Following neoadjuvant therapy, all four patients converted to negative ctDNA prior to surgical resection. The study used the modified Ryan scheme to report tumour histological response after surgical resection. Unfortunately, breakdown by rectal vs. colon cancer was not available for these results. Of the overall colorectal cohort who converted from positive to negative ctDNA after neoadjuvant treatment (total = 18), 12 had a complete or partial pathological response (66.7%). In the cohort with persistently positive ctDNA (total = 5), only one patient (20%) had a complete or partial pathological response. At a median follow-up of 14.5 months, the authors report no difference in recurrence-free survival (RFS) between the two cohorts.

Our final included study was Tan et al., 2025 [18]. This prospective cohort analysis examining the effect of immunotherapy on pMMR colonic tumours enrolled 33 patients with non-metastatic colon cancer. Of these, 31 patients were included in the efficacy analysis. At baseline, 26 patients (84%) had detectable ctDNA. Following neoadjuvant therapy, five patients (19%) demonstrated ctDNA clearance prior to surgery, and all of these corresponded to pathological responders—there was one further pathological responder who was ctDNA + preoperatively. The remaining 19/26 patients (73%) with preoperative positive ctDNA showed no pathological response on histological assessment, with only one patient (5.3%) in this cohort showing a pathological response to neoadjuvant treatment.

### 3.5. Association Between Postneoadjuvant ctDNA Status and Pathological Response

Quantitative pooling of pathological response outcomes was not feasible due to substantial heterogeneity in response definitions, grading systems, and reporting granularity across studies

In the study by Bregni et al., tumour regression was described qualitatively without application of a formal tumour regression grading system, and no discrete categories of pathological complete response (pCR), major pathological response (MPR) or partial response were reported. Seven patients were described as having “some tumour regression” and pathological response was not stratified by ctDNA status. In the cohort reported by Niemann et al., pathological response was assessed using the modified Ryan tumour regression score. However, response categories were aggregated (scores 0–2 reported together as “complete to partial response”), precluding separation of pCR, near-complete, and partial responses. Furthermore, pathological response data were not reported separately for colon versus rectal primaries. In contrast, the prospective cohort by Tan et al. provided discrete, clinically meaningful response categories, defining pCR as 0% residual viable tumour (RVT), MPR as ≤10% RVT, and partial response as ≤50% RVT. Among 31 patients, pCR was observed in 4 (13%)—1 in a patient who was managed non-operatively and the other 3 cases on histological assessment postsurgical resection—MPR in 6 (19%), and partial response in 1 (3%).

Collectively, these differences in response definitions and reporting granularity represent a major source of methodological heterogeneity and precluded quantitative synthesis of pathological response outcomes. Table 3 below summarises the key findings from all three studies.

### 3.6. Evidence Relating to Surgical Omission

Our search found no published studies that formally evaluate the role of ctDNA in guiding surgical omission. Tan et al. describe the case of one patient who achieved a pathological complete response, converted to negative ctDNA, was managed conservatively, and remains disease-free as of their last surveillance. Otherwise, none of the studies included conservative management as an endpoint for complete responders. Although anecdotal, these observations provide early proof of concept supporting the plausibility of using ctDNA to help guide non-operative management in select patients with non-metastatic colon cancer who demonstrate a pathological complete response and lack adverse features.

## 4. Discussion

This systematic review presents the first comprehensive analysis of the potential role of circulating tumour DNA (ctDNA) in guiding surgical omission after neoadjuvant therapy in patients with non-metastatic colon cancer. While increasingly investigated as a biomarker of treatment response and minimal residual disease, its potential role in guiding consideration of non-operative management in colon cancer has not been critically synthesised within a surgical framework previously. By consolidating the available evidence and rigorously examining methodological heterogeneity, response definitions, and outcome reporting, this study clarifies the current stage of evidence development in this field.

Across three included studies comprising 94 patients, our findings demonstrate that ctDNA dynamics during neoadjuvant therapy correlate with pathological response, but current evidence remains insufficient to support the use of ctDNA as a standalone biomarker for surgical decision-making. While ctDNA clearance was consistently observed among patients achieving major pathological responses, the absence of ctDNA clearance strongly predicted residual disease, highlighting the biomarker’s value primarily as a negative predictive tool rather than a definitive indicator for surgical omission. This finding aligns with recent meta-analyses demonstrating that the absence of ctDNA clearance after neoadjuvant immune checkpoint inhibitors has high sensitivity (98%) for predicting the absence of pathological complete response, though with limited specificity (53%) [19].

In the context of colon cancer, the detection rate of baseline ctDNA in our included studies ranged from 42% to 84%, reflecting known variability in ctDNA detectability driven by tumour biology, assay methodology, and disease burden. The studies by Niemann et al. and Tan et al. used tumour-informed next-generation sequencing platforms. In contrast, Bregni et al. employed methylation-based droplet digital PCR, underscoring the heterogeneity in ctDNA assessment approaches that limits cross-study comparisons.

A key finding from our study is that ctDNA clearance does not demonstrate sufficient concordance with pathological complete response to guide surgical omission reliably. Although all pathological responders in the Tan et al. study [18] who cleared ctDNA remained disease-free during follow-up, the converse was not true: ctDNA clearance occurred in patients without a pathological complete response. This limited positive predictive value has been observed across multiple tumour types treated with neoadjuvant immunotherapy, in which approximately 73% of patients achieve ctDNA clearance but only 38% achieve a pathological complete response [19].

The biological basis for this discordance likely reflects several factors. First, ctDNA shedding may be heterogeneous, with viable tumour cells present despite undetectable ctDNA levels, particularly in tumours with lower proliferative rates or those spatially isolated from the vasculature [20]. Second, current ctDNA assays have variable analytical sensitivity, and the threshold for ctDNA “clearance” remains poorly standardised across platforms [19]. Third, micrometastatic disease may persist in regional lymph nodes or distant sites despite clearance of ctDNA derived from the primary tumour, a phenomenon well documented in early-stage colorectal tumours [20,21].

The included studies reflect the evolving landscape of neoadjuvant immunotherapy in colon cancer, with one study (Tan et al.) specifically evaluating pMMR tumours treated with immunotherapy. Although typically preserved in dMMR tumours recent data indicate that a subset of pMMR colon cancers—particularly those with high chromosomal instability and TP53 mutations—may respond to neoadjuvant immune checkpoint blockade, achieving pathological response rates of approximately 26% [18,22]. However, even in this select population, ctDNA clearance alone cannot reliably identify patients suitable for surgical omission, as evidenced by the persistence of residual disease in the majority of ctDNA-positive patients despite immunotherapy exposure.

For dMMR/MSI-H colon cancers, where neoadjuvant immunotherapy achieves pathological complete response rates of 68–95%, the role of ctDNA remains investigational [23,24]. While ctDNA clearance has shown promise in predicting pathological response in dMMR rectal cancer managed with watch-and-wait strategies [25,26], no prospective data currently support this approach in colon cancer, where surveillance strategies and anatomical considerations differ substantially from those in rectal cancer. It is also important to note that no established complete clinical response criteria currently exist for colonic tumours—most studies define this as a pathological response after surgical resection. Still, the question remains, which features should be considered when determining that a colonic tumour has achieved a complete response when the target organ remains in situ? Furthermore, current guidelines also stop short of recommending the use of ctDNA to guide management or surveillance in patients enrolled in rectal cancer watch-and-wait programs [27].

We also noted variation in ctDNA assay platforms employed. Two major platforms were used across our included studies. Tumour-informed assays, which track patient-specific mutations identified from tumour tissue sequencing, generally demonstrate higher sensitivity for minimal residual disease detection than tumour-agnostic approaches [28]. However, tumour-informed assays require adequate tumour tissue, upfront sequencing infrastructure, and longer turnaround times [29], all of which may limit their applicability in the neoadjuvant setting, where treatment decisions need to be made promptly. Methylation-based ctDNA assays, such as the approach used by Bregni et al. [16], offer the advantage of tissue-free detection but may have reduced sensitivity in low-tumour-burden states. The optimal timing of ctDNA assessment also remains uncertain [30,31]. While postneoadjuvant, presurgical sampling was uniformly employed across our included studies, emerging evidence suggests that serial ctDNA monitoring during treatment may provide superior predictive value by capturing dynamic tumour response [32,33].

The strengths of our systematic review include its focused scope on neoadjuvant colon cancer, a comprehensive search strategy, and a synthesis of all available evidence on ctDNA in this clinical context. However, we acknowledge several limitations. The small number of included studies (*n* = 3) and the limited patient cohort (*n* = 94) reflect the nascent state of this field. Substantial heterogeneity in patient populations (MMR status, neoadjuvant treatment regimens), ctDNA assay methodologies, and outcome reporting precluded a formal meta-analysis. Furthermore, no study assessed the feasibility of surgical omission as a primary or secondary endpoint, precluding any formal assessment. The observational nature of all included studies introduces potential selection bias, and the short follow-up duration limits assessment of long-term oncologic outcomes.

We highlight several key research priorities based on our study. First, prospective trials specifically designed to evaluate ctDNA-guided surgical omission in non-metastatic colon cancer are needed—particularly in the setting of dMMR tumours, where several trials are exploring non-operative management across all cancer types that display such mutations [34]. Such trials should incorporate rigorous definitions of clinical complete response, standardised ctDNA assessment platforms with predefined cutoffs for clearance, and acceptable surveillance protocols to detect early regrowth. Second, longer-term follow-up data from existing neoadjuvant immunotherapy trials in colon cancer are essential to understand the durability of pathological responses and the correlation between ctDNA clearance and sustained disease control. Median follow-up periods in current studies remain short, insufficient to capture late recurrences beyond 3 years posttreatment.

Based on the current evidence presented here, ctDNA status alone is not sufficient as a criterion to guide surgical omission in patients with non-metastatic colon cancer after neoadjuvant therapy. While ctDNA clearance may serve as a favourable prognostic indicator and inform discussions about adjuvant therapy intensification or de-escalation in the postoperative setting, the lack of prospective validation, insufficient concordance with pathological complete response, and the potential for occult residual disease preclude its use for surgical decision-making at this time [35]. However, ctDNA may play a role in a multimodal surveillance strategy for select patients with dMMR tumours who achieve complete response—especially given the establishment of many rectal cancer watch-and-wait programs, which may be extrapolated to select colon cancer patients. However, real-world data examining the feasibility of watch-and-wait approaches for colonic tumours are needed first, as are guidelines defining complete response in these patients.

## 5. Conclusions

Current evidence is insufficient to support the use of ctDNA to guide surgical omission. The high negative predictive value of persistently positive ctDNA reliably identifies patients with residual disease. However, ctDNA clearance does not predict pathological complete response with sufficient accuracy to safely forego surgery. Future trials with standardised ctDNA platforms, rigorous surveillance protocols, and adequate follow-up are essential to determine whether ctDNA-guided strategies can safely achieve organ preservation in select patients. Until then, the landscape of colon cancer is likely to remain heavily reliant on surgical resection.

## Figures and Tables

**Table 1:** Study Characteristics.

Study	Study Design	Population	MMR Status	Neoadjuvant Therapy	Pathological Response Grading	Timing of ctDNA Samples	Primary ctDNA-Relevant Outcomes
Bregni et al., 2022 [16]	Prospective phase II trial (retrospective ctDNA analysis)	Stage II–III colon cancer	No differentiation	1 cycle neoadjuvant FOLFOX	Not standardised	Baseline; 2 weeks after NAT; preop (median 20 days after start of NAT)	ctDNA kinetics associated with DFS and OS
Niemann et al., 2024 [17]	Retrospective single-centre cohort	Stage III colon cancer subset	No differentiation	Chemotherapy ± immunotherapy	Modified Ryan score	Baseline pre-NAT; preoperative ctDNA (exact timing not reported)	ctDNA conversion associated with favourable treatment effect score
Tan et al., 2025 [18]	Prospective cohort	Non-metastatic colon cancer	pMMR	Immunotherapy-based neoadjuvant therapy	% residual viable tumour (pCR/MPR)	Baseline pre-NAT; preoperative ctDNA (exact timing not reported)	ctDNA clearance associated with response and recurrence risk

MMR = mismatch repair, pMMR = mismatch repair-proficient, ctDNA = cell tumour DNA, DFS = disease-free survival, OS = overall survival.

**Table 2:** Postneoadjuvant ctDNA status and Pathological Outcomes.

Study	Total Patients (*n*)	Total Number of Eligible Patients Assessed	Baseline ctDNA-Positive	Post-NAT ctDNA-Negative i.e., Converted	MPR or pCR in ctDNA-Negative	MPR or pCR in ctDNA-Positive	Notes
Bregni et al., 2022 [16]	80	60	25/60	NR	NR	NR	ctDNA kinetics correlated with DFS
Niemann et al., 2024 [17]	24	8	4/4	4/4	NR	NR	Converters had favourable pathology
Tan et al., 2025 [18]	31	26	26/31	5/26	5	1	One patient managed non-operatively

**Table 3:** Tumour Response Post Neoadjuvant Treatment by study.

Study	Response Definition	Complete Response (pCR)	Major Pathological Response (MPR)	Partial Response	Notes
Bregni et al., 2022 [16]	Tumour regression reported; no formal TRG categories provided	Not Reported	Not Reported	7 patients with “some tumour regression”	No ctDNA stratified pathology
Niemann et al., 2024 [17] *	Modified Ryan score: 0 = complete, 1 = near complete, 2 = partial, 3 = poor/none	Not reported separately	Not reported separately	Not reported separately	Colon vs. rectum not separately
Tan et al., 2025 [18]	pCR = 0% RVT; MPR ≤ 10% RVT; PR ≤ 50% RVT	4/31 (13%)	6/31 (19%)	1/31 (3%)	Colon only, pMMR cohort

* Note that Niemann et al. did not offer breakdown of complete response/partial response based on tumour stage or location (colon vs. rectum).

## Data Availability

All data generated in this study is from previously published research. The raw data supporting the conclusions of this article will be made available by the authors on request.

## Supplementary Materials

The following supporting information can be downloaded at: https://www.mdpi.com/article/10.3390/cancers18050815/s1. PRISMA 2020 Checklist [36].
